# Course of Treatment and Short-Term Outcome of Surgically Treated Paediatric Upper Limb Fractures during the COVID-19 Pandemic—Experiences of a Level 1 Trauma Centre in Central Europe

**DOI:** 10.3390/children9020172

**Published:** 2022-01-31

**Authors:** Stephan Payr, Theresia Dangl, Andrea Schuller, Philipp Scheider, Britta Chocholka, Manuela Jaindl, Elisabeth Schwendenwein, Thomas M. Tiefenboeck

**Affiliations:** Department of Orthopedics and Trauma Surgery, Division of Trauma Surgery, Medical University of Vienna, Waehringer Guertel 18-20, 1090 Vienna, Austria; theresia.dangl@meduniwien.ac.at (T.D.); n1617891@students.meduniwien.ac.at (A.S.); philipp.scheider@meduniwien.ac.at (P.S.); britta.chocholka@meduniwien.ac.at (B.C.); manuela.jaindl@meduniwien.ac.at (M.J.); elisabeth.schwendenwein@meduniwien.ac.at (E.S.); thomas.tiefenboeck@meduniwien.ac.at (T.M.T.)

**Keywords:** paediatric trauma, fractures, COVID-19, pandemic

## Abstract

The COVID-19 pandemic and the resulting restrictions led to a reduced number of surgeries. This study examines its impact on the course of treatment and clinical outcome of surgically treated paediatric upper limb fractures during that specific period. This retrospective cohort study evaluated all children aged 0–18 years presenting with an upper limb fracture treated surgically at the level 1 trauma centre of the University Clinic of Orthopaedics and Trauma Surgery of Vienna within lockdown from 16 March to 29 May 2020 (definition applied through corresponding legislation) compared to the same period from 2015 to 2019. A total number of 127 children (m:63; f:44) were included. The lockdown did not lead to a significant increase in complications during and after initial surgery. Time until removal of implant was not significantly prolonged (*p* = 0.068; *p* = 0.46). The clinical outcome did not significantly differ compared to previous years. The experience of a level 1 trauma centre showed that despite reduced surgical capacity during the COVID-19 pandemic, no negative differences concerning course of treatment and clinical outcome of surgically treated paediatric upper limb fractures were present. These findings are still of importance since the COVID-19 pandemic continues and several countries in Central Europe are currently under their fourth lockdown.

## 1. Introduction

The COVID-19 outbreak of 2019 was declared a pandemic according to the World Health Organisation (WHO) on 11 March 2020 [1].

Multiple public health measures, including social distancing, wearing masks in public, school closures, the cancellation of sports events and activities, were introduced in countries all over the world including Austria, where lockdown was announced on 16 March 2020 [2,3].

In Austria schools, sports clubs and lido were re-opened, representing the end of the first lockdown, on 29 May 2020 [4]. Such measures were taken to change the epidemiology of patients/children coming to the emergency department [1,5,6,7,8].

During this first lockdown, a shift in medical resources in the outpatient department, reduced capacity for acute and scheduled surgeries and limited appointments at the University Clinic of Orthopaedics and Trauma Surgery, Department of Trauma Surgery were implemented due to COVID-19. Reduced surgical capacity due to the COVID-19 lockdown may have led to an increase in complications after initial surgical treatment in children with upper limb fractures. Further, reduced capacity may have led to a longer time until removal of implant in surgically treated children with upper limb fractures and therefore to a higher complication rate and an impaired outcome after implant removal.

Several observations describe the epidemiological characteristics of injuries—mainly fractures [1,5,8,9]. However, none of these describe how limited medical resources affect treatment and outcome of paediatric fractures.

The objective of this study was to evaluate how the COVID-19 pandemic and the following lockdown from 16 March to 29 May 2020 impacted the treatment regime and complication rates in a single level 1 trauma centre in Central Europe compared to the same period during (from 16 March to 29 May) the past 5 years (2015–2019). We hypothesised that children with surgically treated upper limb fractures experienced a longer time until removal of implant when treated during the first LD in 2020 compared to previous years. Further, we hypothesised that the clinical short-term outcome of children treated during lockdown would not significantly differ from previous years.

## 2. Methods

The Ethics and Scientific Review Committee of the Medical University of Vienna granted ethical approval for this retrospective study on 17 February 2021 (Code: 2315/2020). In this study, all children aged 0–18 years presenting with upper limb fractures were included. They were all treated surgically with K-wires or titanium elastic nails (TENs) at the emergency department (ED) of the University Clinic of Orthopaedics and Trauma Surgery, Department of Trauma Surgery, during lockdown from 16 March to 29 May 2020, and these patients were compared to patients from the same period in 2015–2019.

We defined the Austrian lockdown (16 March to 29 May 2020) as the period during which restrictions were implemented by the government and even monitored by the police. Data were extracted from patient’s charts stored in the information management system (AKIM) of the General Hospital of Vienna.

In children treated with K-wires, implant removal was indicated 6 weeks after initial surgery independent of age. In patients treated with TENs, removal was indicated 3 months after initial surgery for children younger than 6 years and 6 months after initial surgery for children 6 years and older.

### 2.1. General Postoperative Regimen

- Distal radial/antebrachial fractures and supracondylar fractures (K-wires): Discharge on day 2, forearm cast for 3 weeks, removal of stiches 12–14 days after surgery. Radiographs are taken after two weeks, after cast removal and prior removal of implant. Clinical investigation is performed two weeks after cast removal and if a deficit is still present, two weeks after removal of implant and on a regular basis. Regular appointments are scheduled to discover growth disorders early on.
- Antebrachial fractures (TENs): Discharge on day 2, forearm cast for 3 weeks, and removal of stiches 12–14 days after surgery.

Children younger than 6 years (TENs for 3 months): Radiographs are taken after two weeks, after cast removal and in week 8.

Children 6 years and older (TENs for 6 months): Radiographs are taken after two weeks, after cast removal and in week 8. If bony union is not present, radiographs are taken every four weeks until bony union is achieved.

For all children, clinical investigation is performed two weeks after cast removal and if deficits are present, children are investigated clinically on a regular basis.

Demographic variables (sex and patients’ age at injury) were collected. Patients’ upper limb fractures were categorised whether treated with K-wires or TENs. Treated patients were further categorised according to age as mentioned above. Time until removal of implant (ROI) in days was assessed for the first hypothesis. Complications of surgery according to Sink et al.’s [10] classification, number of scheduled appointments until ROI and clinical deficits at last follow-up were assessed for the second hypothesis.

Further parameters such as time until presentation in days, time until surgery (in days), length of hospitalisation (in days), unscheduled appointments until ROI, number of X-rays until ROI, the occurrence of radiologic deterioration (deficit) of the fracture, time until complication and mean follow-up (FUP) are also included. Classification according to Sink et al. [10]: (I) a complication that requires no treatment and has no clinical relevance; (II) a deviation from the normal postoperative course that requires outpatient treatment; (III) a complication that requires surgical or an unplanned hospital admission; (IV) a complication that is life threatening, requires ICU admission, or is not treatable without the risk of permanent disability; and (V) death [10]. In order to assess every patient, grade I was therefore considered as “no complication”. Injuries that occurred during the COVID-19 outbreak restrictions are further grouped as a cohort referred to as the “lockdown” (LD) group. This group was compared to injuries that presented in the 2015–2019 study window referred to as the “pre-lockdown” (pre-LD) group.

The comparative period of five years was chosen to reduce potential bias by having an adequate control sample size representing a steady baseline. Furthermore, during these years, no similar restrictions were implemented at any time, making this period adequate for the control group.

**Figure 1:**
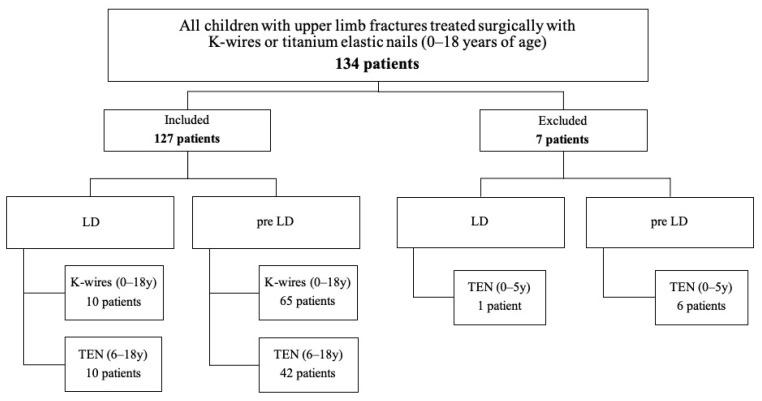
Flowchart of study population.

Due to the low number of children under the age of 6 that were treated surgically with TENs (LD: 1 patient; pre-LD: 1.2 patients), further statistical analysis of this cohort of patients was not performed. After applying the inclusion and exclusion criteria, 127 of an initial 134 children were included (Figure 1). For statistical evaluation, only children treated with K-wires and children over 6 years treated with TENs were included, resulting in 20 patients, m:12 f:8, in the LD period of 2020 and 107 patients (mean: 21.4 ± 5.6; mean m:13.8 ± 1.8, f:7.6 ± 3.5) in the pre-LD period of 2015–2019 with surgically treated fractures of the upper limb.

### 2.2. Statistical Analysis

Descriptive data (mean ± SD) are reported for the entire patient cohort. Differences between means and proportions were tested with the Fisher exact test for categorical variables, the unpaired *t*-test for continuous variables, and the Mann–Whitney-U test when not normally distributed. Normal distribution was tested using the Shapiro–Wilk test. Statistical significance was set at a level of *p* < 0.05. Microsoft Excel and GraphPad software version 6.00, (GraphPad Software, San Diego, CA, USA) were used for statistical evaluation.

## 3. Results

Surgical capacity at the department was reduced by 54.6% during LD compared to the mean surgical capacity of previous years (LD: 350 surgeries; pre-LD: 770.4 ± 41.6 surgeries).

The average patient during LD was slightly younger in both cohorts—the K-wire cohort (LD: 10 children, m: 6, f: 4; mean age 5.9 ± 3.7 years vs. pre-LD: 13 ± 2.5 children m:8.6; f:4.4; mean age 7.3 ± 3.2 years) and the TENs cohort (LD: 10 children, m:7, f:3; mean age 8.2 ± 2.4 years vs. pre-LD: 8.4 ± 3.1 children, m:5.2, f:3.2; mean age 9.6 ± 2.9 years). Comparing the LD and pre-LD periods, there was no difference in time until presentation, time until surgery and the length of stay at the hospital. All variables and *p*-values of this investigation are summarised in Table 1 and Table 2.

### 3.1. Surgical Complications According to Sink et al.’s Classification

#### 3.1.1. Children Treated with K-Wires (LD vs. Pre-LD)

Of 10 patients who were treated during LD, only one patient had a complication of grade III and 9 were considered grade I. This one complication was present 15 days after initial surgery and early implant removal was necessary.

The mean number of patients who developed a complication according to Sink et al. [10] in the pre-LD period was 1 ± 1 (5 in total in the entire control observation period—ECOP) of 13 ± 2.5 patients. There were 0.6 ± 0.5 grade II and 0.4 ± 0.5 grade III (3 Gr. II and 2 Gr. III in the ECOP) complications and 12 ± 3.2 patients had no complications, classed as grade I.

The mean time until complication was 30 ± 11.3 days after initial surgery and early implant removal was necessary for 0.4 ± 0.5 (2 patients in the ECOP).

#### 3.1.2. Children (≥6 Years) Treated with TENs (LD vs. Pre-LD)

Of the 10 patients who were treated during LD, no patients experienced complications, classed as grade I.

The mean number of patients who developed a complication according to Sink et al. [10] in the pre-LD period was 8.4 ± 3.1 of 1 ± 0.7 patients (5 in the ECOP). There were 0.6 ± 0.9 grade II and 0.4 ± 0.5 grade III (3 Gr. II and 2 Gr. III in the ECOP) complications and 7.4 ± 3 (37 patients in ECOP) had no complications, classed as grade I. The mean time until complication was 106 ± 71 days after initial surgery and early implant removal was necessary for 0.4 ± 0.5 (2 patients in the ECOP).

### 3.2. Time Until Removal of Metal Implant

#### 3.2.1. Children Treated with K-Wires (LD vs. Pre-LD)

In total, in 10 children during LD, implant removal was performed 79.6 ± 44.5 days after primary surgery. In the pre-LD period, implant removal was performed in 13 ± 2.5 children after 62.37 ± 31.8 days (*p* = 0.068).

#### 3.2.2. Children (≥6 Years) Treated with TENs

Comparing the time until removal in 10 children in LD (154.5 ± 38.5 days) with that of 8.4 ± 3.1 children in the pre-LD period (156.5 ± 53.6 days), there was no statistical difference (*p* = 0.46).

During and after implant removal, no surgery related complications were present in patients treated in the LD and pre-LD periods.

### 3.3. Follow-Up Treatment

Mean time to follow-up treatment in the K-wires group in the LD period was 95.2 ± 55.9 days and 81.9 ± 93.1 days in the pre-LD period; mean time to follow-up treatment in the TENs group of children ≥6 years in the LD period was 163.1 ± 40.9 days; and 167.9 ± 55.6 days in the pre-LD period; *p* = 0.399. The number of scheduled/unscheduled appointments and the number of performed radiographs did not indicate any difference in the groups between observation periods.

### 3.4. Outcome

#### 3.4.1. Children Treated with K-Wires

LD: One patient revealed an impairment in radiological outcome; and after FUP, two patients presented with clinical deficits (an extension deficit of 5° and a flexion deficit of 15°).

Pre-LD: Radiological deterioration was observed in 1 ± 0.7 (5 patients in ECOP) and clinical deficits in 2 ± 2 (10 patients in ECOP).

#### 3.4.2. Children (≥6 Years) Treated with TENs

LD: No radiological deterioration or clinical deficits were observed.

Pre-LD: Radiological deterioration was observed in 0.2 ± 0.4 (1 patient in ECOP) and clinical deficits in 0.5 ± 0.5 (10 patients in ECOP).

## 4. Discussion

This manuscript reveals that despite reduced capacity (over 50%) because of the COVID-19 pandemic restrictions, no negative effects were detected concerning the treatment of paediatric upper limb fractures in a level 1 trauma centre in Central Europe.

First, the COVID-19 pandemic did not lead to delayed presentation of paediatric patients when a fracture of the upper limb was suspected. Further, despite strict guidelines concerning preparation for surgery, this did not delay the necessary surgeries.

Most importantly, during and after initial surgery, the number of complications documented did not increase compared to in the pre-LD period. During follow-up, it was also shown that despite reduced capacity, time until removal of implant was not significantly different (K-wires approx. 6 weeks; TENs in children ≥6 years approx. 6 months). All in all, follow-up (appointments and radiographs) and clinical outcome did not differ from usual. One might argue that the restrictions (closure of schools and less outdoor activities) on paediatric patients may have impacted surgical complications. From experience, children that sustain an injury that requires surgery are usually more careful in the post-op course and need to regain confidence in the healed extremity in order to start participating in activities. Therefore, the authors do not think that restricted activities during lockdown beneficially affected the outcome of complications compared to previous years. Further, the steady baseline of previous years reveals one complication per year, as in the LD period.

A reduction in surgeries during the COVID-19 pandemic is described in other studies as well [11,12]. The rather immediate presentation and surgery on the day of injury is concomitant to literature [1,11].

To date, the literature mainly focus on paediatric fractures during the COVID-19 pandemic [1,5,8,13,14,15]. The herein presented study is the first study in the current literature analysing clinical outcome and possible adverse effects of surgically treated paediatric upper limb fractures during the LD with a concomitant reduction in surgical capacity. Karia et al. describe the effect of the COVID-19 pandemic as a reduction in the number of surgeries and state that prioritisation is needed, but do not comment on clinical outcomes [12]. Simon et al. suggest that complications were low but lacks follow-up on patients to actually confirm their observation [16]. 

During follow-up in this present study, no reduction in scheduled appointments and radiographs taken was detected. A reason for this could be that a further reduction in appointments at the outpatient department was not completely implemented on such a short time and therefore a difference is not detectable within this period of time. Another explanation might be that because of the fact that our department represents the largest paediatric trauma department in Austria, patients’ appointments were already limited to a minimum because of the large number of patients in general, meaning that a further reduction because of the COVID-19 pandemic was not necessary or possible to implement.

Overall, this study has several limitations such as the retrospective single-centre design, leading to only *n* = 10 patients included in the LD period. This is a small number to conclude from compared to 65 children in the previous 5 years in the same period of time, although that is a mean of 13 patients per year (in the pre-LD period) and thus comparable to the LD period. However, the data presented are from one of the largest university hospitals worldwide and the main paediatric trauma centre in Austria. Given this fact, paediatric trauma patients were especially centralised towards our department during LD, making it questionable if a multicentral study design would be the solution if taking possible differences in resources, expertise and regimens into account. In this study, only surgically treated (K-wires and TENs) upper limb fractures were evaluated. These injuries are the most common fractures in the paediatric population and are therefore representative [16]. Combining these fractures into one category seems adequate for this study since it also has an epidemiological character. Further, we do not compare the fractures per se, their treatment and their severity/range of deficits, but only describe the occurrence in general. Further, the surgery is relatively similar and the follow-up protocol, as described, is relatively the same at our department.

A distinct advantage is the longer period of comparison (five years) and associated large number of patients in this study, minimising bias by having a steady baseline and resembling this consistency compared to current literature (and, respectively, the longer period of comparison than in other studies) [1,5,6,8,14,15,17]. As hypothesised, the course of treatment and outcome did not significantly differ due to the COVID-19 restrictions at this level 1 trauma centre, the most representative paediatric trauma department in Austria, despite the low number of patients included in the LD cohort. Many studies compare their data to the most recent year, which makes their cohort, and thus their results, relatively susceptible to bias [6,7,18,19]. This study further gives an accurate overview of surgically treated paediatric upper limb fractures from a level 1 trauma centre contrary to the majority of current data from tertiary trauma centres [1,5,8,14,15].

## 5. Conclusions

The experience of a level 1 trauma centre in Central Europe showed that despite reduced surgical capacity during the COVID-19 pandemic and the following imposed restrictions by the Austrian government, no negative differences concerning course of treatment and clinical outcome of surgically treated paediatric upper limb fractures were present. These findings are still of importance since the COVID-19 pandemic continues and several countries in Central Europe are currently under their fourth lockdown.

## Figures and Tables

**Table 1:** Comparison of LD and pre-LD periods with presentation of *p*-values (N.A. = not applicable).

	K-Wires			TENs (≥6 Years)		
	LD (Total)	Pre-LD (Mean)	*p* =	LD (Total)	Pre-LD (Mean)	*p* =
Complications after initial surgery	1	1 ± 1	N. A.	0	1 ± 0.7	N. A.
*t* until ROI (in days)	79.6 ± 44.5	62.37 ± 31.8	0.068	154.5 ± 38.5	156.5 ± 53.6	0.46
Scheduled appointments until ROI	3.3 ± 1.1	4 ± 1	0.126	4.6 ± 0.7	4.9 ± 1.7	0.617
Clinical deficit	2	2 ± 2	N. A.	-	0.5 ± 0.5	N. A.

**Table 2:** Supplementary data describing the LD and pre-LD periods.

	K-Wires		TENs (≥6 Years)	
	LD (Total)	Pre-LD (Mean)	LD (Total)	Pre-LD (Mean)
*n* =	10	13 ± 2.5	10	8 ± 3.1
Age	5.9 ± 3.7	7.3 ± 3.2	8.2 ± 2.4	9.6 ± 2.9
*t* until presentation (in days)	0 ± 0	0.02 ± 3.2	0.1 ± 0.3	0.26 ± 1.1
*t* until surgery (in days)	0.2 ± 0.4	0.12 ± 0.8	0.4 ± 0.7	0.14 ± 0.6
Length of stay (in days)	2 ± 0.7	1.89 ± 1.3	1.9 ± 0.6	1.79 ± 0.6
Unscheduled appointments until ROI	0.2 ± 0.4	0.25 ± 0.5	0.2 ± 0.4	0.29 ± 0.6
Number of x-rays until ROI	2.5 ± 0.7	3.2 ± 0.8	3.8 ± 0.6	4 ± 1.1
Radiologic deficit	1	1 ± 0.7	-	1 ± 0.4
*t* until complication (in days)	15	30 ± 11.3	-	100.2 ± 71
Mean follow-up (in days)	95.2 ± 55.9	81.9 ± 93.1	163.1 ± 40.9	167.9 ± 55.6

## Data Availability

The datasets generated and/or analysed in the current study are not publicly available due to data privacy but are available from the corresponding author on reasonable request.
